# Multiple Sclerosis Susceptibility Genes: Associations with Relapse Severity and Recovery

**DOI:** 10.1371/journal.pone.0075416

**Published:** 2013-10-09

**Authors:** Ellen M. Mowry, Robert F. Carey, Maria R. Blasco, Jean Pelletier, Pierre Duquette, Pablo Villoslada, Irina Malikova, Elaine Roger, R. Phillip Kinkel, Jamie McDonald, Peter Bacchetti, Emmanuelle Waubant

**Affiliations:** 1 Multiple Sclerosis Center, Department of Neurology, Johns Hopkins University, Baltimore, Maryland, United States of America; 2 Multiple Sclerosis Center, Department of Neurology, University of California San Francisco, San Francisco, California, United States of America; 3 Department of Neurology, Hospital Universitario Puerta de Hierro, Madrid, Spain; 4 Pole de Neurosciences Cliniques, Service de Neurologie, Centre de Résonance Magnétique Biologique et Médicale, Centre Hospitalier Universitaire Timone, Aix Marseille Université, Marseille, France; 5 Multiple Sclerosis Clinic, Centre Hospitalier de L′Universite de Montreal, Montreal, Canada; 6 Center of Neuroimmunology, Institute of Biomedical Research August Pi Sunyer-Hospital Clinic, Barcelona, Spain; 7 Department of Neurology, Beth Israel Deaconess Medical Center, Harvard Medical School, Boston, Massachusetts, United States of America; 8 Department of Epidemiology and Biostatistics, University of California San Francisco, San Francisco, California, United States of America; University of Oxford, United Kingdom

## Abstract

**Objective:**

Patients with early multiple sclerosis (MS) have stereotyped attack severity and recovery. We sought to determine if polymorphisms in MS susceptibility genes are associated with these attack features or with the risk of a second attack.

**Methods:**

503 white subjects evaluated within a year of MS onset were included in the study. The severity of and recovery from the first two attacks were determined based on published definitions. Seventeen MS susceptibility genes were genotyped at the UCSF MS Genetics laboratory. Each polymorphism was evaluated in multivariate ordinal models, adjusted for the other polymorphisms, for its association with attack severity and recovery. We also assessed if these polymorphisms were associated with increased risk of a second attack.

**Results:**

The *MPHOSPH9* polymorphism was associated with greater attack severity (odds ratios [OR] = 1.47, 95% CI [1.11, 1.94], p = 0.008), while the *RGS1* and *TNFRSF1A* polymorphisms tended to be associated with reduced attack severity. The *CD6* polymorphism tended to be associated with increased odds of worse attack recovery (OR = 1.25, 95% CI [0.93, 1.68], p = 0.13). In those who were *HLA-DRB1*-negative, the *EVI5* polymorphism was associated with attacks of less severity; in *HLA-DRB1* positive patients, EVI5 was associated with attacks of greater severity and worse recovery. The *IL7R*, *TNFRSF1A*, and *GPC5* polymorphisms tended to be associated with having a second event within a year.

**Conclusions:**

Some MS susceptibility polymorphisms may be associated with attack severity, recovery, or frequency. Further characterization of these genes may lead to a better understanding of MS pathogenesis and to a more individualized treatment approach.

## Introduction

While the severity of and recovery from multiple sclerosis (MS) attacks vary substantially from one person to the next, an individual patient's early attacks are likely to be of stereotyped severity and recovery [1]. In other words, a person who has a severe first attack is at greater risk of a severe second attack, and a person who has poor neurologic recovery from the first attack is more likely to recover poorly from the second attack.

In the past few years, the number of genes that have been confirmed as important to MS susceptibility has increased substantially [2]–[13]. Whether these genes are associated with the clinical phenotype of the disease is less clear. In this preliminary investigation, we sought to determine if genetic polymorphisms associated with MS susceptibility are associated with the severity of and recovery from early attacks of MS or with the risk of a second attack.

## Methods

### Ethics statement

This study was approved by the University of California, San Francisco (UCSF) Committee on Human Research (CHR); each center contributed data from patients enrolled in Institutional Review Board-approved studies who had provided written informed consent. The only study/institution in which minors were enrolled was at UCSF; in the case of all minors, verbal assent was provided by the patient and written informed consent was provided by a parent.

### Subjects and sites

White subjects with clinically isolated syndrome (CIS) or relapsing-remitting MS at five MS centers and from two clinical trials who were followed prospectively from within a year of disease onset and had brain magnetic resonance imaging (MRI) within six months of MS onset. At UCSF, three cohorts were used. From the UCSF MS Center, data for all children and adult patients seen within one year of MS onset are prospectively collected [1]. Clinic visits usually occur every 6 months, and unscheduled visits occur if a patient has an exacerbation. Subjects from the atorvastatin or riluzole trials at UCSF were also included [14], [15]. Atorvastatin trial participants were seen within 90 days of onset and were followed monthly for the first three months and then every three months for 18 months; unscheduled visits occurred for relapses. For the riluzole study, which was incomplete at the time of the data freeze, patients were enrolled within a year of disease onset and were seen monthly for the first six months, then every three months for two to three years; additional visits were scheduled for relapses. At the MS Unit of the Department of Neurology in Marseille, France, data from a cohort of patients seen within six months of disease onset who participated in a prospective natural history study of MS that began in 2000 were captured using the EDMUS database [16]. Clinic visits and MRIs were typically scheduled every 3 months during the first year, every 6 months for the next 3 years, and every year subsequently. Patients from the Centre Hospitalier de L′Université de Montreal, Canada who met the inclusion/exclusion criteria were offered participation in the study from September, 2007 until March, 2009. Patients were seen every year or more regularly if the disease was active. Subjects seen at the University of Navarra, Spain who were seen at MS onset were enrolled in a prospective biomarker study of MS beginning in 2001 [17]. Subjects were followed at the clinic every 3 to 6 months after MS onset. Clinical and demographic data were collected prospectively using the EDMUS database. At the Hospital Universitario Puerta de Hierro, subjects with CIS seen within one year of onset underwent neurologic examination at three months and were followed every six months unless they had a relapse, in which case they were seen for an unscheduled visit. The Optic Neuritis Treatment Trial (ONTT) was a multicenter study that enrolled subjects within the first 8 days of optic neuritis; some had had prior attacks [18]. For purposes of the current study, those without prior neurologic symptoms consistent with a previous MS/CIS attack were excluded. Follow-ups occurred on days 4, 15, and 30, weeks 7, 13, and 19, months 6 and 12, and then yearly [19]. The Controlled High Risk Avonex Multiple Sclerosis Prevention Study in Ongoing Neurologic Surveillance (CHAMPIONS) study was an open-label extension of Controlled High Risk Subjects Avonex Multiple Sclerosis Prevention Study (CHAMPS) [20], [21]. CHAMPS enrolled subjects without a prior episode of neurologic dysfunction who were seen within 27 days of the onset of neurologic symptoms consistent with MS and had an abnormal brain MRI (≥2 clinically silent MS-consistent lesions). Subjects were randomized to weekly intramuscular interferon beta-1a versus placebo. CHAMPIONS enrolled willing CHAMPS participants for ongoing open-label follow-up. Subjects were seen every 6 months in both studies; unscheduled relapse assessments occurred within seven days of new symptom onset for the CHAMPS phase of the study, or within 2 weeks in the extension phase.

### Outcomes

Relapses were defined as new or recurring neurological symptoms referable to the CNS lasting for at least 48 hours after a remission of 30 days or more since the previous attack in the absence of fever or known infection. Pseudoexacerbations were excluded. The severity of and recovery from the first attack were determined based on definitions derived from previous publications [1]. Mild first attack severity was defined as Functional Systems (FS) scores of 0 to 1 in one to three FSs, or visual acuity (VA) better than or equal to 20/40, EDSS score 0 to 1.5 inclusive; moderate severity was defined as a score of at least 2 but not higher than 2 in one or two FSs or four or more scores of 1 or VA of 20/50 to 20/190, EDSS 2.0 to 2.5 inclusive; severe was assigned for relapses exceeding prior criteria. Recovery was scored using the lowest EDSS and FS scores between two to 12 months after the attack. For the first attack, recovery was considered complete (no residual complaint, normal follow-up examination, all FS scores = 0, follow-up EDSS score = 0), fair (residual subjective complaint that does not impair activity, or at least one FS score of 1 at most or VA better or equal to 20/40, follow-up EDSS = 1.0 to 1.5), or poor (at least one FS score of 2 or more or VA of 20/50 or worse).

For the second event, severity was scored the same way if the pre-event EDSS was 0. When the second event was preceded by incomplete recovery, the severity was defined as mild (EDSS increase by 0.5 point, or 1 point change in up to three FS scores), moderate (EDSS increase by 1 or 2 points, or 2 points change in up to two FS, or 1 point change in four or more FS), or severe (exceeding prior criteria). Recovery from the second event was defined as complete if no residual signs or symptoms remained above those which were present prior to the attack, fair if EDSS increased by up to one point or if there was an increase of one point on one or two FS (e.g. residual subjective complaint or new residual finding compared to baseline that does not impair activity), or poor if exceeding prior criteria.

### Predictors: genetic polymorphisms

Several genetic polymorphisms outside of the HLA region have been validated as being associated with the risk of developing MS [2]–[13]. For this study, in addition to *HLA-DRB1*, we selected the 16 non-HLA genes that had been validated as of 2010 (Table 1). Genotyping was conducted in the laboratory of Dr. Jorge Oksenberg with TaqMan SNP genotyping assays (Applied Biosystems Inc., Foster City, CA, USA). The following were contained in every PCR reaction: 10 ng of DNA, 1×TaqMan Genotyping Master Mix, and 1×SNP assay (both from Applied Biosystems, Inc.). An ABI 97000 GeneAmp PCR system (Applied Biosystems, Inc.) was used for amplification. The following PCR program was used: 95° Celsius for 10 minutes, then 50 cycles of 95° Celsius for 15 seconds and 62° Celsius for one minute. An ABI prism 7900HT Sequence Detection System (with SDS 2. software; Applied Biosystems, Inc.) was used to read the plates. A PCR locus-specific amplification was used for *DRB1*, as previously described [9].

**Table 1:** Non-HLA genes associated with multiple sclerosis tested in this study.

Gene	Single Nucleotide Polymorphism	Minor Allele (Major Allele)	Number (%) of subjects with one or two copies of minor allele	Risk Allele for MS
*CD58* [4], [6], [10]	rs2300747	G (A)	94 (19)	A
*RGS1* [6], [12]	rs2760524	A (G)	150 (30)	G
*EVI5* [2], [9]	rs10735781	C (G)	419 (83)	G
*KIF21B* [3]	rs12122721	A (G)	250 (50)	G
*IL12A* [6], [12]	rs4680534	C (T)	302 (60)	C
*TMEM39A* [3]	rs1132200	A (G)	115 (23)	G
*IL7R* [5], [6], [10]	rs6897932	T (C)	210 (42)	C
*IL2RA* [2], [6], [7], [10]	rs2104286	G (A)	184 (37)	A
*CD6* [6]	rs17824933	G (C)	233 (46)	G
*MPHOSPH9* [12]	rs1790100	G (T)	217 (43)	G
*TNFRSF1A* [6]	rs4149584	T (C)	41 (8)	T
*CLEC16A* [9], [11]	rs12708716	G (A)	286 (57)	A
*CD226a* [9], [11]	rs763361	C (T)	354 (70)	T
*TYK2* [5], [9]	rs34536443	C (G)	25 (5)	G
*IRF8* [6]	rs17445836	A (G)	165 (33)	G
*GPC5* [8]	rs727986	C (T)	203 (40)	C

### Statistics

We dichotomized each gene as having one or two versus no copies of the risk allele and assessed its association with severity, recovery, and risk of a second event. We also looked for interactions (defined by *p* value for interaction term<0.1) between *HLA-DRB1* and *EVI5* and *HLA-DRB1* and *CD226a* because such interactions have been identified in MS susceptibility studies [7], [9]. All genes were studied in multivariate models to assess each polymorphism's effect independent of the others.

Because severity and recovery were measured on an ordered, three-level scale, predictors of these attack features were analyzed with repeated measures ordinal logistic regression; we used the Stata ologit command, with the “vce (cluster)” option. We attempted to confirm the results by assessing the outcomes as dichotomous using generalized estimating equations with robust standard errors. To determine the association of polymorphisms with second attack risk, we used a multivariate Cox proportional hazards model. The exact date of the second attack could not be established in most of the ONTT patients who had a second attack, although a range of possible second attack dates was available. As such, to approximate an approach from a prior publication [22], we also conducted a multivariate logistic regression analysis, adjusted for length of follow-up, in which the outcome was the binary outcome of a second attack within 1 year (which could be ascertained for all but four patients).

## Results

### Patient and attack characteristics

We identified 503 patients (n = 199 from UCSF; n = 43 from Marseille; n = 36 from Montreal; n = 13 from Pamplona; n = 60 from Madrid; n = 68 from CHAMPIONS; n = 84 from ONTT). The mean age at onset was 33±9 years; 359 (71%) were women. Initial brain MRI showed abnormalities consistent with MS in 459 (91%). Genotyping failure occurred only for *IRF8* in one patient. All subjects had at least one copy of the *TYK2* G allele, so we assessed if two G alleles versus one was associated with the outcomes. Similarly, no subject had more than one copy of the *TNFRSF1A* T allele, so we assessed if one versus no copies of the T allele was associated with the outcomes. Nearly half the patients (n = 244; 49%) had one or more copies of HLA-DRB1*1501. Allele frequencies for the non-*HLA* polymorphisms are presented in Table 1.

A second attack was experienced by 69% (n = 349); mean follow-up was 6.5±5.0 years in those who did not have a second attack. DMT was initiated in 69% (n = 345) during the follow-up period, but only 134 (27%) had received DMT for at least 90 days immediately preceding the second attack or, if no second attack occurred, preceding the end of follow-up. Relapse severity and recovery are presented in Table 2.

**Table 2:** Attack severity and recovery.

Event Characteristic	First Event	Second Event*
Severity, n (%)**		
Mild	226 (45)	128 (47)
Moderate	192 (38)	95 (35)
Severe	85 (17)	48 (18)
Recovery, n (%)***		
Complete	282 (58)	151 (56.3)
Fair	151 (31)	84 (31.3)
Poor	54 (11)	33 (12.3)

* While 349 in the overall cohort had a second event during follow-up, of those in the CHAMPIONS and ONTT cohorts (total n = 152, 104 (68%) of whom had a second attack), second attack severity was not calculable for ONTT patients and was calculable for 28 (61%) of CHAMPIONS patients who had a second attack. Second attack recovery was calculable for 34 CHAMPIONS patients and no ONTT patient; ** could not be characterized for 2 second events among non-ONTT/non-CHAMPIONS cohorts; *** could not be characterized for 16 first attacks overall or for 11 second events among non-ONTT/non-CHAMPIONS cohorts.

### Genetic predictors of attack severity

The associations of the polymorphisms with attack severity are presented in Table 3. The *MPHOSPH9* risk allele was associated with increased odds of more severe events (odds ratio [OR] = 1.47, 95% confidence interval [CI] [1.11, 1.94], p = 0.008), while the *RGS1* (OR = 0.47, 95% CI [0.21, 1.03], p = 0.060) and *TNFRSF1A* (OR = 0.63, 95% [0.40, 1.00]), p = 0.050) polymorphisms were associated with tendencies for reduced attack severity. The univariate results were similar (Table S1 in File S1), and the results were not meaningfully different when use of DMT prior to the attack (or end of the follow-up, when no second attack occurred), was included in the models (data not shown). When event severity was dichotomized, there was an additional trend for *IL12A* to be associated with an attack being moderate or severe (Table S2 in File S1). When a more extreme outcome (severe versus mild or moderate attacks) was assessed (Table S2 in File S1), there was additionally a strong association between the *CD58* polymorphism and reduced odds of a severe attack (OR = 0.25, 95% CI [0.06, 0.95], p = 0.042). Further, *HLA-DRB1* and *IL2RA* tended to be associated with attacks being severe.

**Table 3:** Association of MS susceptibility genes with attack severity and recovery (multivariate models).

Gene	More severe attacks	Worse recovery from attacks
*RGS1*	0.47 (0.21, 1.03), p = 0.060	0.96 (0.29, 3.18), p = 0.94
*CD6*	1.01 (0.76, 1.33), p = 0.96	1.25 (0.93, 1.68), p = 0.13
*MPHOSPH9*	**1.47 (1.11, 1.94), p = 0.008**	1.05 (0.78, 1.41), p = 0.77
*TNFRSF1A*	0.63 (0.40, 1.00), p = 0.050	0.77 (0.44, 1.34), p = 0.35
*HLA-DRB1*	0.95 (0.71, 1.28), p = 0.74	1.10 (0.82, 1.48), p = 0.52
*CD58*	0.43 (0.10, 1.84), p = 0.25	0.54 (0.11, 2.64), p = 0.45
*EVI5*	0.97 (0.72, 1.30), p = 0.83	1.22 (0.90, 1.65), p = 0.20
*KIF21B*	1.04 (0.58, 1.87), p = 0.89	1.11 (0.50, 2.48), p = 0.80
*IL12A*	1.16 (0.87, 1.55), p = 0.32	1.03 (0.77, 1.37), p = 0.85
*TMEM39A*	0.82 (0.21, 3.24), p = 0.78	0.90 (0.39, 2.11), p = 0.82
*IL7R*	0.91 (0.48, 1.73), p = 0.77	0.80 (0.41, 1.59), p = 0.53
*IL2RA*	1.19 (0.63, 2.26), p = 0.59	1.38 (0.61, 3.14), p = 0.44
*CLEC16A*	1.28 (0.79, 2.09), p = 0.32	0.99 (0.64, 1.52), p = 0.96
*CD226a*	1.09 (0.78, 1.53), p = 0.60	1.08 (0.78, 1.51), p = 0.64
*TYK2*	0.86 (0.50, 1.48), p = 0.59	1.00 (0.50, 2.01), p = 0.99
*IRF8*	0.94 (0.38, 2.31), p = 0.89	0.62 (0.24, 1.61), p = 0.32
*GPC5*	1.03 (0.77, 1.39), p = 0.83	1.06 (0.79, 1.44), p = 0.69

Results are presented as odds ratios (95% confidence intervals), p values.

There was an interaction of *HLA-DRB1* and *EVI5* (p = 0.004 for interaction term). Among those who were *HLA-DRB1* negative, *EVI5* was associated with reduced odds of a more severe attack (OR = 0.62, 95% CI [0.43, 0.91], p = 0.015) but tended to have greater odds of a more severe attack in the *HLA-DRB1*-positive group (OR = 1.54, 95% CI [0.97, 2.45], p = 0.068). There was no strong evidence for interaction between *HLA-DRB1* and *CD226a* (p =  0.44 for interaction term). The results were similar in models in which the odds of severe/moderate attacks were ascertained, although when severe attacks were the outcome, the p value for the interaction term between *HLA-DRB1* and *CD226a* was 0.060. *CD226a* was associated with lower odds of severe attacks in *HLA-DRB1*-negative patients (OR = 0.56, 95% CI [0.30, 1.05], p = 0.07), while it did not appear to be meaningfully associated with severity in *HLA-DRB1*-positive patients (OR = 1.34, 95% CI [0.70, 2.54], p = 0.38).

### Genetic predictors of attack recovery

Only the *CD6* polymorphism tended to be associated with worse attack recovery (OR = 1.25, 95% CI [0.93, 1.68], p = 0.13), although the CIs included 1.0 (Table 3). The results were similar in univariate models (Table S1 in File S1) or when recovery was dichotomized into incomplete versus complete (Table S2 in File S1), or when DMT use was added into the multivariate models (data not shown).

There was an interaction of *HLA-DRB1* and *EVI5* in the attack recovery model (p = 0.011 for interaction term). Among those who were *HLA-DRB1* negative, *EVI5* did not appear to be substantially associated with recovery (OR = 0.82, 95% CI [0.54, 1.24], p = 0.34), but it was associated with greater odds of worse recovery in the *HLA-DRB1*-positive group (OR = 1.80, 95% CI [1.16, 2.81], p = 0.009); results were similar when incomplete recovery was the outcome. There was no strong evidence for interaction between *CD226a* and *HLA* in either recovery model (p value for interaction term = 0.37 for primary model; p = 0.61 for incomplete recovery model).

### Genetic predictors of second event risk

Overall, none of the polymorphisms of interest appeared to be strongly associated with second attack risk, although the CIs were wide (Table 4). However, there were trends of interest: the *TNFRSF1A* polymorphism was associated with increased attack risk, while the *CD58* polymorphism was associated with decreased risk. There was no strong evidence for interaction between *HLA-DRB1* and *EVI5* (p value for interaction term = 0.27) or *CD226a* (p = 0.75). When the odds of a second attack occurring within a year were evaluated, *TNFRSF1A, IL7R,* and *GPC5* tended to be associated with increased odds thereof (Table 4). The results were similar in univariate models (Table S3 in File S1), with *IL12A* demonstrating an association with slightly reduced odds of a second attack within a year.

**Table 4:** Association of MS susceptibility genes with occurrence of second event (multivariate models).

Gene	Second event (OR) within a year*	Risk (HR) of second event**
*CD58*	0.66 (0.17, 2.63), p = 0.56	0.53 (0.26, 1.09), p = 0.082
*IL7R*	1.88 (0.73, 4.88), p = 0.19	1.41 (0.82, 2.41), p = 0.22
*TNFRSF1A*	1.61 (0.82, 3.18), p = 0.17	1.29 (0.88, 1.90), p = 0.19
*GPC5*	1.31 (0.88, 1.95), p = 0.19	0.95 (0.75, 1.20), p = 0.65
*HLA-DRB1*	1.10 (0.74, 1.62), p = 0.65	1.02 (0.81, 1.29), p = 0.88
*RGS1*	0.57 (0.16, 1.96), p = 0.37	0.90 (0.39, 2.06), p = 0.80
*EVI5*	1.09 (0.72, 1.64), p = 0.69	1.08 (0.85, 1.38), p = 0.53
*KIF21B*	1.02 (0.44, 2.34), p = 0.96	0.97 (0.59, 1.60), p = 0.92
*IL12A*	0.77 (0.52, 1.15), p = 0.20	1.03 (0.82, 1.31), p = 0.78
*TMEM39A*	0.56 (0.12, 2.71), p = 0.47	1.17 (0.47, 2.91), p = 0.74
*IL2RA*	0.81 (0.31, 2.14), p = 0.67	1.05 (0.60, 1.85), p = 0.87
*CD6*	0.82 (0.55, 1.21), p = 0.31	1.04 (0.82, 1.31), p = 0.77
*MPHOSPH9*	0.89 (0.60, 1.32), p = 0.56	0.99 (0.78, 1.24), p = 0.91
*CLEC16A*	1.20 (0.63, 2.31), p = 0.58	0.80 (0.55, 1.16), p = 0.24
*CD226a*	1.00 (0.64, 1.54), p = 0.99	1.02 (0.79, 1.32), p = 0.88
*TYK2*	1.14 (0.46, 2.86), p = 0.78	1.14 (0.67, 1.93), p = 0.63
*IRF8*	1.33 (0.41, 4.32), p = 0.63	1.28 (0.67, 2.45), p = 0.45

* missing 4 and **44 ONTT patients.

Results are presented as odds ratios (ORs) or hazard ratios (HRs) with 95% confidence intervals, p values.

## Discussion

The variability in outcomes between patients with MS leads to prognostic uncertainty, which causes significant psychological stress for patients and their caregivers [23], [24]. It also makes the role of the physician more difficult in that for an individual patient, a plan of care must be made using group-level data. With the advent of more potent but more risky DMTs for MS, developing better models for predicting prognosis is imperative so that those who are unlikely to have bad disease outcomes are not exposed unnecessarily to hazardous therapies. Genetic polymorphisms associated with MS are attractive candidate predictors for inclusion in prognostic models. Other studies have evaluated the association of MS risk alleles with the rate of MS progression or risk of conversion from CIS to RRMS and have found little evidence of such associations [25], [26].

Herein, we have the first preliminary evidence that some of the genes known to increase MS risk may also be associated with the severity of early attacks or the degree of recovery from them, although the wide confidence intervals suggest that the without confirmation in a second, larger dataset, the associations should not be over-interpreted. Nonetheless, from a mechanistic standpoint, the results are intriguing. *MPHOSPH9*, which has unknown function [12], was associated with greater attack severity. One hypothesis regarding its function is that this SNP is associated with reduced expression of the nearby *CDK2AP1* gene, a cell cycle regulator [27]. *RGS1* proteins terminate G-protein signaling and reduce lymphocyte trafficking; *in vitro* and *in vivo*, they are regulated in part by interferon beta-1b, an MS therapy [28]. It is thus biologically plausible that the *RGS1* risk allele may influence attack severity. The *TNFRSFA1* polymorphism, associated with a tendency for less severe early attacks, is of functional interest because the TNFα pathway has been implied in MS pathogenesis. TNFα inhibitors, used to treat some autoimmune disorders, are thought to precipitate demyelinating episodes consistent with MS [6]. There were several genes that had possible relationships with attack severity when an extreme phenotype (severe versus mild/moderate attacks) was the outcome. The most convincing relationship was for *CD58*, a CD2 ligand expressed on T-cells that is important to their differentiation and proliferation [4], [10]. The *CD58* A allele confers greater risk of MS; however, having one or more copies of the allele was associated with mild or moderate attacks. This result is somewhat surprising in that *CD58* expression, which is increased in remission compared to relapse, is greater in those with the protective G allele [4]. While these results need to be confirmed, it may be that the *CD58* has different roles in establishing MS risk or relapse timing and in influencing the severity of relapses. The gene product of CD6, which tended to be associated with worse recovery, is involved in continuing the activation of T cells; in healthy individuals with two copies of the MS risk allele, CD4+ T cell proliferation is actually reduced [29].

Of interest *EVI5*, which is associated with lymphoma development [13], appeared to be associated with event severity and recovery, but the direction of the association differed depending on a person's *HLA-DRB1* status; those who were negative for *HLA-DRB1* had events characterized by less severity, while those who were positive for *HLA-DRB1* had events of greater severity or worse recovery. The directions of these associations are similar to those reported for MS susceptibility; this finding highlights the complexity of analyzing the effects of polymorphisms considering the likelihood of many unidentified interactions between them [7], [9].

None of the polymorphisms assessed showed a convincing association with risk of early second attack or of a second attack overall, although the confidence intervals were too wide to provide evidence against possibly substantial associations.

The study has some limitations. We restricted the analysis to white patients to reduce heterogeneity, which reduces generalizability to other racial groups. We included those evaluated within a year of onset to minimize recall bias and the inclusion of pseudoexacerbations in the analysis. However, this decision could have led to inclusion bias, as subjects who present later could be different from those who come to medical attention earlier. While fairly objective, our definitions of severity and recovery still rely on the EDSS (or its components), which has its own limitations in precision and responsiveness [30], [31]. That the ONTT and CHAMPIONS datasets were missing information relevant to the study may have introduced bias in addition to increasing uncertainty. Because our assessment of these genes was hypothesis-driven and since the study was an exploratory investigation that was not intended to prove with finality any associations, we did not correct for multiple comparisons. Rather than focusing only on p values, we were interested in evaluating the directions and magnitudes of the effects seen in the context of the plausible biological connections between the SNPs and MS. Thus, our results need replication in an independent, larger dataset. It would also be valuable to evaluate SNPs identified subsequent to the genotyping done for this study for their association with the severity, recovery, and risk of second attacks [25].

Our results support prioritizing investigations of the identified genes to confirm the associations, to better characterize their role in MS pathogenesis, to determine if they may provide new therapeutic targets, and to help guide the use of DMT at the individual patient level.

## Supporting Information

File S1 **Supporting information.** Table S1. Association of MS susceptibility polymorphisms with attack severity and recovery (univariate models). Table S2. Association of risk alleles with dichotomous severity/recovery (multivariate models). Table S3. Association of MS susceptibility genes with second event (univariate models).
(DOCX)
