# Supplementary material for: Multiple Sclerosis Susceptibility Genes: Associations with Relapse Severity and Recovery
Source: PLoS One. 2013 Oct 9;8(10):e75416. doi: 10.1371/journal.pone.0075416 (PMC3793991; doi:10.1371/journal.pone.0075416)
Supplement: File S1 — Supporting information. Table S1. Association of MS susceptibility polymorphisms with attack severity and recovery (univariate models). Table S2. Association of risk alleles with dichotomous severity/recovery (multivariate models). Table S3. Association of MS susceptibility genes with second event (univariate models). (DOCX) [file pone.0075416.s001.docx]

Table S1. Association of MS susceptibility polymorphisms with attack severity and recovery (univariate models)

| Gene | More severe attacks | Worse recovery from attacks |
| --- | --- | --- |
| *HLA-DRB1* | 0.94 (0.71, 1.25), p=0.68 | 1.10 (0.82, 1.46), p=0.54 |
| *CD58* | 0.41 (0.09, 1.91), p=0.26 | 0.60 (0.13, 2.84), p=0.52 |
| *RGS1* | 0.49 (0.20, 1.19), p=0.12 | 0.94 (0.30, 2.92), p=0.92 |
| *EVI5* | 0.91 (0.68, 1.22), p=0.54 | 1.19 (0.88, 1.61), p=0.27 |
| *KIF21B* | 1.02 (0.58, 1.79), p=0.95 | 1.07 (0.48, 2.36), p=0.87 |
| *IL12A* | 1.15 (0.86, 1.53), p=0.36 | 1.05 (0.78, 1.41), p=0.76 |
| *TMEM39A* | 0.80 (0.22, 2.93), p=0.74 | 0.83 (0.36, 1.89), p=0.65 |
| *IL7R* | 1.08 (0.57, 2.03), p=0.81 | 0.86 (0.45, 1.64), p=0.65 |
| *IL2RA* | 1.20 (0.65, 2.20), p=0.56 | 1.31 (0.60, 2.84), p=0.50 |
| *CD6* | 1.02 (0.77, 1.35), p=0.88 | 1.26 (0.94, 1.68), p=0.12 |
| *MPHOSPH9* | **1.46 (1.10, 1.93), p=0.009** | 1.00 (0.75, 1.34), p=0.97 |
| *TNFRSF1A* | 0.65 (0.42, 1.02), p=0.060 | 0.78 (0.46, 1.34), p=0.37 |
| *CLEC16A* | 1.23 (0.78, 1.94), p=0.38 | 0.98 (0.65, 1.48), p=0.93 |
| *CD226a* | 1.12 (0.81, 1.56), p=0.49 | 1.11 (0.80, 1.54), p=0.54 |
| *TYK2* | 0.81 (0.47, 1.41), p=0.45 | 0.99 (0.50, 1.97), p=0.97 |
| *IRF8* | 0.94 (0.40, 2.24), p=0.89 | 0.60 (0.23, 1.56), p=0.29 |
| *GPC5* | 1.04 (0.78, 1.39), p=0.79 | 1.04 (0.78, 1.40), p=0.78 |

Results are presented as odds ratios (95% confidence intervals), p values

Table S2. Association of risk alleles with dichotomous severity/recovery (multivariate models)

| Gene | Moderate/Severe vs. Mild Attacks | Severe vs. Mild/Moderate Attacks | Incomplete Attack Recovery |
| --- | --- | --- | --- |
| *HLA-DRB1* | 0.85 (0.62, 1.15), p=0.29 | 1.34 (0.89, 2.01), p=0.16 | 1.09 (0.81, 1.46), p=0.57 |
| *CD58* | 0.74 (0.24, 2.25), p=0.59 | 0.25 (0.06, 0.95), p=0.042 | 0.77 (0.22, 2.72), p=0.68 |
| *RGS1* | 0.37 (0.11, 1.26), p=0.11 | 0.53 (0.20, 1.41), p=0.21 | 0.99 (0.33, 3.01), p=0.99 |
| *EVI5* | 0.92 (0.66, 1.27), p=0.60 | 1.15 (0.77, 1.74), p=0.49 | 1.18 (0.86, 1.62), p=0.30 |
| *KIF21B* | 0.96 (0.50, 1.85), p=0.91 | 1.10 (0.47, 2.55), p=0.83 | 1.23 (0.61, 2.49), p=0.56 |
| *IL12A* | 1.29 (0.95, 1.75), p=0.10 | 0.88 (0.59, 1.31), p=0.53 | 1.02 (0.76, 1.36), p=0.92 |
| *TMEM39A* | 0.75 (0.18, 3.11), p=0.69 | 0.89 (0.18, 4.31), p=0.88 | 0.66 (0.20, 2.19), p=0.50 |
| *IL7R* | 0.93 (0.47, 1.82), p=0.83 | 0.82 (0.35, 1.91), p=0.65 | 0.82 (0.40, 1.66), p=0.58 |
| *IL2RA* | 1.04 (0.48, 2.24), p=0.92 | 2.22 (0.71, 6.93), p=0.17 | 1.44 (0.70, 2.99), p=0.32 |
| *CD6* | 0.96 (0.71, 1.29), p=0.77 | 1.07 (0.72, 1.57), p=0.75 | 1.26 (0.94, 1.70), p=0.12 |
| *MPHOSPH9* | 1.48 (1.10, 2.00), p=0.010 | 1.45 (0.99, 2.13), p=0.058 | 1.04 (0.77, 1.40), p=0.82 |
| *TNFRSF1A* | 0.67 (0.40, 1.12), p=0.13 | 0.48 (0.22, 1.08), p=0.076 | 0.77 (0.44, 1.36), p=0.37 |
| *CLEC16A* | 1.17 (0.69, 2.00), p=0.57 | 1.67 (0.77, 3.63), p=0.20 | 0.93 (0.61, 1.43), p=0.75 |
| *CD226a* | 1.20 (0.86, 1.69), p=0.28 | 0.87 (0.56, 1.35), p=0.54 | 1.07 (0.76, 1.49), p=0.70 |
| *TYK2* | 0.72 (0.39, 1.33), p=0.29 | 1.12 (0.44, 2.89), p=0.81 | 1.08 (0.56, 2.07), p=0.82 |
| *IRF8* | 0.95 (0.38, 2.39), p=0.91 | 0.94 (0.28, 3.15), p=0.93 | 0.69 (0.30, 1.61), p=0.40 |
| *GPC5* | 0.97 (0.71, 1.32), p=0.83 | 1.19 (0.80, 1.78), p=0.39 | 1.06 (0.78, 1.43), p=0.72 |

Results are presented as odds ratios (95% confidence intervals), p values

Table S3. Association of MS susceptibility genes with second event

(univariate models)

| Gene | Second event within a year (OR)* | Risk (HR) of second event** |
| --- | --- | --- |
| *HLA-DRB1* | 1.09 (0.75, 1.59), p=0.64 | 0.98 (0.78, 1.23), p=0.86 |
| *CD58* | 0.58 (0.15, 2.20), p=0.43 | 0.55 (0.27, 1.11), p=0.096 |
| *RGS1* | 0.56 (0.17, 1.86), p=0.34 | 0.88 (0.39, 1.98), p=0.75 |
| *EVI5* | 1.05 (0.70, 1.56), p=0.82 | 1.07 (0.85, 1.36), p=0.55 |
| *KIF21B* | 1.11 (0.50, 2.48), p=0.80 | 1.03 (0.63, 1.68), p=0.92 |
| *IL12A* | 0.77 (0.53, 1.13), p=0.18 | 1.01 (0.81, 1.27), p=0.91 |
| *TMEM39A* | 0.62 (0.14, 2.83), p=0.54 | 1.31 (0.54, 3.20), p=0.55 |
| *IL7R* | 1.69 (0.67, 4.29), p=0.27 | 1.40 (0.82, 2.39), p=0.22 |
| *IL2RA* | 0.94 (0.37, 2.38), p=0.90 | 1.06 (0.61, 1.84), p=0.85 |
| *CD6* | 0.83 (0.57, 1.21), p=0.33 | 1.03 (0.82, 1.29), p=0.82 |
| *MPHOSPH9* | 1.01 (0.69, 1.48), p=0.94 | 0.98 (0.78, 1.23), p=0.89 |
| *TNFRSF1A* | 1.56 (0.81, 2.99), p=0.18 | 1.24 (0.85, 1.80), p=0.26 |
| *CLEC16A* | 1.15 (0.61, 2.17), p=0.67 | 0.81 (0.57, 1.17), p=0.26 |
| *CD226a* | 1.01 (0.66, 1.54), p=0.96 | 1.01 (0.79, 1.30), p=0.92 |
| *TYK2* | 1.23 (0.50, 3.00), p=0.66 | 1.19 (0.71, 2.01), p=0.50 |
| *IRF8* | 1.44 (0.46, 4.52), p=0.54 | 1.27 (0.67, 2.38), p=0.46 |
| *GPC5* | 1.33 (0.91, 1.95), p=0.14 | 0.95 (0.75, 1.20), p=0.66 |

*missing 4 and **44 ONTT patients

Results are presented as odds ratios (ORs) or hazard ratios (HRs) with 95% confidence intervals, p values
